# Physicochemical, Microbiological, and Sensorial Quality Attributes of a Fermented Milk Drink (Laban) Fortified with Date Syrup (Dibs) during Cold Storage

**DOI:** 10.3390/foods10123157

**Published:** 2021-12-20

**Authors:** Abdullah M. Alhamdan, Fahad Y. Al Juhaimi, Bakri H. Hassan, Kheled A. Ehmed, Isam A. Mohamed Ahmed

**Affiliations:** 1 Chair of Dates Industry & Technology, College of Food & Agricultural Sciences, King Saud University, Riyadh 11451, Saudi Arabia; alhamdan@ksu.edu.sa (A.M.A.); kehmed@ksu.edu.sa (K.A.E.); 2 Department of Food Science and Nutrition, College of Food & Agricultural Sciences, King Saud University, Riyadh 11451, Saudi Arabia; faljuhaimi@ksu.edu.sa; 3 Department of Agricultural Engineering, College of Food & Agricultural Sciences, King Saud University, Riyadh 11451, Saudi Arabia; bakri@ksu.edu.sa

**Keywords:** laban (fermented milk), date dibs, nutritional quality, sensory, shelf-life

## Abstract

This study investigated the nutritional, microbial, and sensory quality attributes of a fermented milk (laban) drink flavored with date syrup (dibs) during cold storage at 4 °C for 7 days. Date syrup was added to laban in specific proportions (2.5, 5, 7.5, 10, 12.5, and 15% date syrup/total weight of flavored laban) and an appropriate percentage (12.5%, 74 °Bx) was selected based on the sensory preference of panelists. The results indicate that flavoring laban with date syrup affected the physicochemical, nutritional, microbial, and sensory quality attributes of the product in different ways. Incorporation of date syrup in fresh laban drink significantly increased the pH, ash, protein, total solids, sugars, and magnesium (*p* < 0.05). However, acidity, fat, casein, lactose, calcium, total microbial count, and total yeast and molds count were decreased (*p* < 0.05). During storage, acidity, ash, and microbial load were concomitantly increased, while fat, casein, total solids, and sugars showed a concurrent reduction as the storage period progressed. The panelists preferred the freshly prepared flavored laban drink compared with the stored one, which is not surprising. After 7 days of storage, flavored laban drink was more acceptable than a non-flavored one. The findings of this research will help in fortifying dairy products with dates to create highly nutritious drinks without the addition of artificial additives, refined sweeteners, and preservatives, which at the same time would be accepted by consumers.

## 1. Introduction

Date palm (*Phoenix dactylifera* L.) is a multipurpose and highly climate-tolerant plant whose cultivation and utilization has widely expanded all over the world. Saudi Arabia is the second top world producer of dates and produced about 1.54 million tons of dates in 2019 from a 118 thousand hectare harvested area [1]. Dates are one of the most popular fruits in the world, and they are considered to be a good source of dietary fiber, glucose, fructose, potassium, magnesium, selenium, non-starch polysaccharides, and a wide variety of bioactive compounds, and consequently they possess antioxidant, antimicrobial, anticancer, anti-inflammatory, and anti-infertility activity [2,3]. In addition to direct consumption of fresh dates, they are processed into numerous products such as powder, syrup, juice, jam, relish, pickles, vinegar, and alcohol [4]. Of these products, date syrup (dibs) is the most common processed date product produced from the surplus of dates in Saudi Arabia. Date dibs contains high amounts of glucose and fructose, potassium, calcium, sodium, magnesium, citric, acetic, malic, oxalic, and succinic acids and possesses antioxidant and antihemolytic activity [5,6]. Thus, the utilization of date syrup as a flavoring agent and sweetener in food products is of high importance from both nutritional and health standpoints.

The dairy industry is one of the most advanced food processing sectors in Saudi Arabia that has achieved high growth in recent years. In Saudi Arabia, dairy production from cows, sheep, camels, and goats was increased from 0.8 million tons in 1999 to 2.7 million tons in 2019 [1]. Accordingly, several types of dairy products such as pasteurized milk, sterilized milk, yogurt, cheese, and flavored milk and fermented milk (laban) products were produced and consumed in Saudi Arabia [7].

Laban is an important fermented dairy beverage that is produced traditionally and industrially in many African, Middle Eastern, and Southeast Asian countries [8]. It is produced from both raw and pasteurized milk through lactic acid fermentation either spontaneously or by the application of starter culture of *Streptococcus thermophilus*, *Lactobacillus acidophilus*, and *Lactobacillus delbrueckii* subsp. *bulgaricus* [9]. Laban is considered as a vital dairy drink because of its low calorie and fat content, in addition to its probiotic potential [8,10]. A metagenomic study on laban showed the presence of numerous genes of vitamins and essential amino acids metabolism and synthesis, suggesting the high nutritional value of this dairy beverage [10]. 

Most of the dairy products available in the markets contain several flavoring agents—namely, chocolate, caramel, coffee, vanilla, strawberry, and banana—and they also contain refined sugars and preservatives [11,12,13]. These products achieved high popularity in recent years as nutritious, tasty, and functional foods, and there is still increased demand for dairy products with improved nutritional and health quality attributes. In this regard, date dibs has been found to improve the nutritional, functional, microbial, and sensory quality attributes of whey beverage [14], probiotic ice cream [15], date juice milk drink [16], and flavored and probiotic yogurt [17,18]. In addition to the above-mentioned utilization of date dibs in dairy products, it could also be used as a safe and nutritious flavoring material for developing flavored fermented milk drink. Therefore, this study was conducted to investigate the effect of date syrup (dibs) on the nutritional, microbial, and sensory quality attributes of laban (fermented milk) drink, as well as its shelf life during cold storage.

## 2. Materials and Methods

### 2.1. Materials

Fermented cows’ milk (laban) was obtained from a giant dairy processing plant in the Riyadh region, Saudi Arabia, on the same production day. Date syrup of the Khalas variety was obtained from a date factory in the Alhasa region, Saudi Arabia. All chemicals utilized in this study were obtained from Sigma-Aldrich (Sigma, St. Louis, MO, USA).

### 2.2. Preparation of Laban Drink

Laban drinks were prepared by mixing laban samples and different concentrations of syrup (2.5, 5.0, 7.5, 10.0, 12.5, and 15.0 g syrup/100 g laban drink) using a Coldream M2 mixing apparatus (Italy) at 5 °C for 5 min. The samples were subjected to sensory evaluation by 36 semi-trained panelists, and the best concentration was selected as 12.5 g syrup/100 g laban, 74 °Bx. After that, three drink samples were prepared as follows: non-fortified laban drink that was considered as a negative control (NC), laban drink flavored with sugar solution (glucose and fructose, 74° Bx) and considered as a positive control (PC), and laban drink flavored with 12.5 g date syrup/100 g laban, 74 °Bx (DSL). Then, laban drink samples were stored in sealed sterilized polyethylene bottles at 4 °C, where the nutritional and microbial quality attributes of the products were assessed daily for the 7 days of storage.

### 2.3. Determination of Total Soluble Solids and Water Activity

The total solids in laban samples and date syrup were measured using an ABBA5 refractometer (BS instruments, Jena, Germany). Water activity of laban samples and date syrup was measured using Aqualab model series (Decagon Devices, Inc. Pullman, Washington, DC, USA) following ISO 18,787 method [19].

### 2.4. Color Analysis

The color attributes of date syrup and laban samples were determined using a Color Flex Hunter Lab system (Lab Scan XE, Reston, VA, USA) as described by Alhamdan et al. [20]. Prior to analysis, calibration of the colorimeter was carried out using a standard white plate. Then, the CIE1932 was used to measure the color attributes: L* (lightness to darkness, 100 to 0), a* (redness to greenness, 0 to 100 = red; −80 to 0 = green), and b* (yellowness and blueness, 0 to 70 = yellow; −100 to 0 = blue) of triplicate samples date syrup and laban.

### 2.5. Determination of pH

The pH of the syrup and laban drink samples was assessed using a Jenway pH meter Model 3510 (Jenway, Stone, UK). 

### 2.6. Chemical Composition

The AOAC standard official methods were used for the determination of crude protein, crude fat, ash, acidity, casein content. The crude protein content was measured using the Kjeldahl method [21] and using 6.38 as conversion factor. The crude fat was determined using the Gerber method [22]. Ash content was measured using the ignition method [21]. The acidity was determined using the titration method [21]. The casein was determined by measuring the total nitrogen and the non-casein nitrogen using the standard methods [21].

### 2.7. Total Energy

The total energy was calculated by multiplying the protein and carbohydrate with the factor 4 and fat with the factor 9.

### 2.8. Determination of Lactose Contents

The lactose content of the samples was measured using the spectrophotometric method at 520 nm as described by Teles et al. [23].

### 2.9. Determination of Sugar Contents

The concentration of reduced sugar (glucose and fructose) and non-reduced (sucrose) sugars was measured using the HPLC method [21]. The sample (5 μL) was injected into a NH2-Supelcosil (Supelco Inc., Bellefonte, PA, USA) LC column (250 mm × 4.6 mm, ID, 5 μm particle size) attached to a 10AD Shimadzu HPLC system (Shimadzu Corporation, Kyoto, Japan). The sample was run at 2.5 mL/min using a gradient of solution A (20% water) and B (80% acetonitrile), and the results were compared and quantified using the results of authentic sugar standards (Sigma Chemical Co., St. Louis, MO, USA) run in the same way as with the samples. 

### 2.10. Determination of Minerals and Vitamins

The mineral content of the samples was measured using the Atomic absorption methods, as described by Singh et al. [24], after ashing the samples at 550 °C and digestion with a mixture of concentrated nitric and hydrochloric acids. Vitamins were extracted from the samples by mixing 10 mL laban with 10 mL ethanol and then homogenizing by sonication for 5 min. After that, three extraction steps were performed using a mixture of hexane and dichloromethane (9:1, *v*/*v*), and the extract was dried under nitrogen. After dissolving it in 1 mL ethanol, analysis of vitamins was carried out using GC-MS, as described by Kadioglu et al. [25].

### 2.11. Determination of Microbial Counts

The microbial counts of bacteria, mold, and yeasts were determined as described by Kunadu et al. [26] and expressed as CFU/g. The plate count agar (Oxoid, Hampshire, UK) was used for bacterial count, and the total counts were measured after incubation at 37 °C for 1–2 days. Potato dextrose agar was used for yeast and mold count, and the media was incubated at 25 °C for 3–7 days.

### 2.12. Sensory Evaluation

The sensory attributes of laban drinks were assessed using a 9-point hedonic scale (1 = extremely dislike and 9 = extremely like) in two sessions. Prior to the analysis, three training sessions were conducted with 36 panelists (male, age 30–61 years) of the staff of the College of Food and Agricultural Sciences, King Saud University (Riyadh, Saudi Arabia). The panelists were selected based on their interest and skill in sensory assessment and were coached to be expert in the evaluation of the color, taste, consistency, flavor, and sweetness of laban drink samples with and without date dibs. The training was adapted to achieve understanding of the sensory parameter measurement scope and sensory borderlines, and this was achieved via individual testing, followed by group discussions. The sensory tests of each sample were conducted properly in a sensory assessment room equipped with separated cabinets and individual lightning at 20 ± 2.0 °C. The samples were coded with three-digit numbers and served randomly to the panelists, and the mean scores of each sample and session were calculated and analyzed.

### 2.13. Statistical Analysis

The data of three independent experiments were collected and statistically analyzed using SPSS software (SPSS, Chicago, IL, USA). One-way analysis of variance was used to determine the significance of the difference between the means and Duncan’s multiple range tests were used to separate the means at *p* < 0.05.

## 3. Results and Discussion

### 3.1. Physicochemical Composition of Raw Materials (Date Dibs and Laban)

The main raw materials (date syrup and laban) were analyzed prior to the development of the syrup-flavored laban drink, and the results are shown in Table 1. Date syrup contains substantial amounts of sugars, mainly glucose and fructose, minerals (e.g., calcium, sodium, and magnesium), vitamin D, and energy. Laban also contains high amounts of minerals, mainly calcium, potassium, sodium, and magnesium. Moisture, water activity, protein, fat, and lightness values were higher in laban than syrup, whereas ash, acidity, pH, and redness values were higher in syrup than laban. The results on physicochemical composition of laban are partially comparable with those reported for different types of laban produced traditionally or industrially in different countries [27,28,29,30]. There are several factors that explain the variations in the physicochemical composition of date syrup and laban. The chemical composition of laban depends on various factors such as type and age of the cows, lactation season, time and stage, feed type and composition, type of starter culture, incubation time, and environmental conditions [9,27,31]. Factors such as date palm cultivar, age, agronomical practices, maturity stage, harvest time and seasons, environmental conditions, and dibs processing conditions also affect the physicochemical composition of syrup [32,33]. The combination of date syrup and laban in the developed products could lead to a product with improved physicochemical attributes.

### 3.2. Sensory Analysis of Laban Drink Fortified with Different Concentrations of Date Syrup

**Figure 1:**
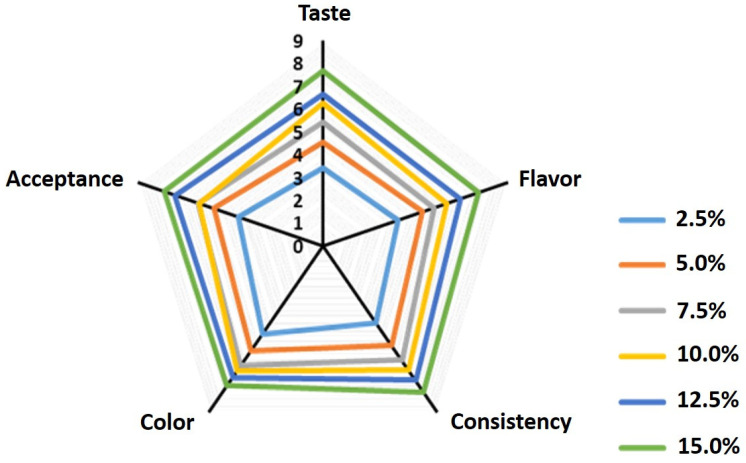
Sensory preference of laban drink fortified with different concentrations (2.5, 5.0, 7.5, 10.0, 12.5, and 15.0%) of date syrup.

Sensory evaluation of laban supplemented with different levels (2.5, 5.0, 7.5, 10.0, 12.5, and 15%) of date syrup was carried out to select the best percentage of the syrup to be used for the development of date syrup-flavored laban drink (DSL). The results indicate that laban fortified with high levels of date syrup (12.5 and 15.0%) showed higher scores on all sensory attributes compared with those fortified with low levels of date dibs (Figure 1). These results indicate that increasing the levels of the date syrup in the product greatly improved its sensory attributes. Similarly, a previous report indicated that increasing the percentage of dibs in yogurt significantly increased the flavor, texture, and overall acceptance of the product [33]. Despite the high preference of laban drink fortified with 15% syrup, it was not significantly different from the drink fortified with 12.5%. Therefore, the later was selected for the development of laban drinks as lower amounts (12.5%) of date syrup were used and hence might be of lower cost than that when using 15% syrup.

### 3.3. Chemical Composition of Date Syrup-Fortified Laban Drink (DSL) during Cold Storage

The changes in the chemical composition of NC, PC, and DSL drinks during cold storage are shown in Table 2. The incorporation of date syrup in laban drink significantly increased (*p* < 0.05) the ash, protein, and total solids content compared with that in NC and PC samples. The increment in ash, protein, and total solids content of laban drink following the addition of date syrup is likely due to the high ash and total solids content of dibs (Table 1). Similar results on the increment in ash, total solids, and protein following the addition of 2% dibs to fermented milk products were also reported previously [33]. In addition, incorporation of different concentrations (9%, 20% and 30%) of date fruits into yogurt formulations increased protein, ash, and total solids content of the products [34]. Moreover, date palm paste increased the levels of total solids and ash in fermented beverage of camel and goat milk [35], while date extract did not affect the ash, protein, and fat content of fermented milk drink [36]. Variations between these studies could be attributed to the differences in milk source, fermentation process conditions, fermentation types, fermenting microbes, and final product types and conditions.

The acidity and fat were significantly lower (*p* < 0.05) in DSL than NC and PC, suggesting that the incorporation of syrup can negatively influence the fat and acidity levels of laban drink. The difference in acidity among laban samples (NC, PC, and DSL) could be due to variations in the microbial composition, which resulted from different carbon and nitrogen sources in the three samples [37]. Similarly, previous a report indicated that the addition of 2% dibs affected the fat and acidity levels of yogurt in a negative manner [33]. In addition, a reduction in fat content was observed following the addition of dates to fermented milk beverage [35,36] and the addition of date syrup to processed cheese [38].

The storage also affected the chemical composition in different ways. Ash and acidity content was significantly increased (*p* < 0.05) as the storage period progressed, whereas fat and casein levels showed a significant reduction (*p* < 0.05) as the storage time was increased. The increase in the acidity of laban drinks during storage could be attributed to the continuous hydrolysis of sugars by fermenting microbes and liberation of organic acids, which increased the acidity of the products [33,37]. The reduction in fat and casein during the storage of laban samples is likely due to the degradation of fat and casein with microbial lipases and proteases, respectively [33,39]. Similar observations on the increase in acidity and ash and reduction in fat and casein during cold storage were also reported [33,37,39,40].

### 3.4. Sugar Content of Date Syrup-Fortified Laban Drink (DSL) during Cold Storage

**Figure 2:**
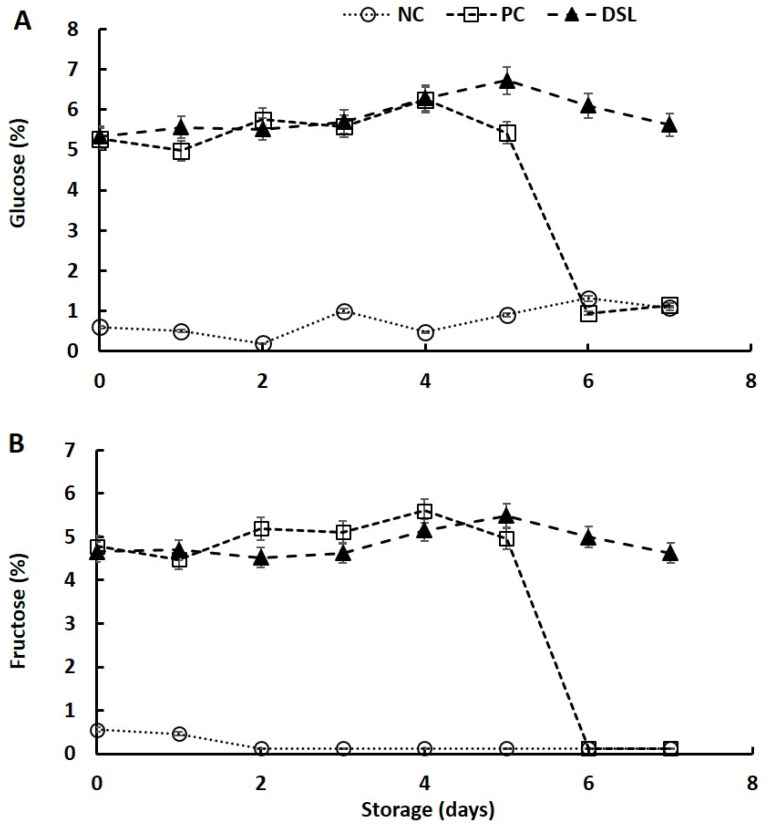
Changes in glucose (**A**) and fructose (**B**) content of date syrup-fortified laban (DSL), positive control (PC), and negative control (NC) drinks during cold storage at 4 °C for 7 days.

Initially, the sugar content in control (NC and PC) and DSL was analyzed at 0 day of storage, and the results indicated that incorporation of syrup in laban drink significantly increased (*p* < 0.05) the levels of total sugars and reduced the lactose content (data not shown). The results on the effect of flavoring laban with 12.5% syrup on the sugar content of the product during cold storage for 7 days are shown in Figure 2. The addition of 12.5% date syrup (DSL) and sugar solution (PC) significantly increased (*p* < 0.05) the fructose and glucose content of the laban drink compared with that of non-fortified product (NC), which could be due to the high content of glucose and fructose in the syrup and sugar solution. The glucose content of NC fluctuated during storage, with a general increase at the end of the storage period. The glucose content of PC was slightly increased to the maximum at day 4 of storage and thereafter sharply reduced as the storage progressed. The glucose content of DSL generally remained constant during storage, with slight increase at the fifth day of storage (Figure 2A). Fructose content of NC rapidly reduced to minimum levels at the second day of the storage and then remained constant as the storage progressed, whereas that of PC fluctuated during the first 5 days of storage and then sharply reduced as the storage time increased (Figure 2B). The fructose content of DSL remained almost constant during storage, with slight increase at day 5 of storage. High reduction in fructose in non-fortified laban drink during storage could be attributed to the low concentration of fructose in this sample and the absence of other sugars such as glucose, which is highly preferred for fermentation by microorganisms [41]. Fluctuation of fructose and glucose content during storage of laban drink fortified with date syrup was likely due to the effects of two contradicting factors: (a) formation of fructose and glucose during hydrolysis of sucrose in the syrup by the fermenting microorganism and (b) further degradation of formed fructose and glucose by the microorganism. Variations in the microbial genera in DSL and its capability for hydrolysis of sucrose, glucose, and fructose directly affected the fructose and glucose content of the product [42,43,44]. In addition, the stability of glucose content during storage of DSL could be ascribed to the antimicrobial effects of phenolic compounds in date syrup, which can inhibit or reduce the metabolic activity of fermenting microbes [45,46]. In agreement with these findings, previous studies indicated that type of added materials and storage period influenced the fructose and glucose content of fermented milk samples and attributed that to the variations in the fermenting microbial consortium and sugar composition of added materials [42,43,44].

### 3.5. Mineral Content of Date Syrup-Fortified Laban Drink (DSL) during Cold Storage

**Figure 3:**
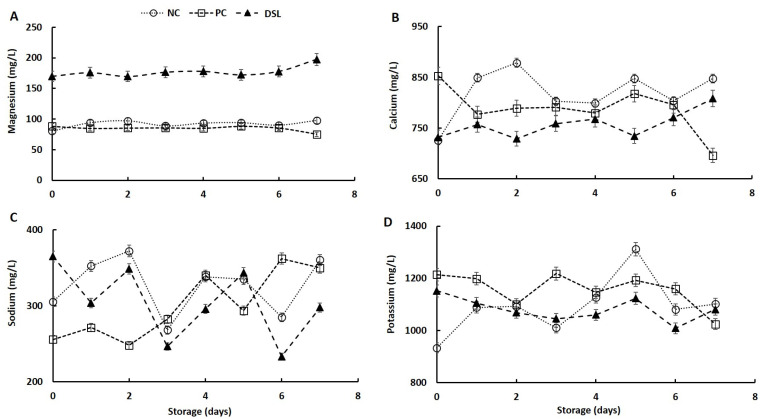
Changes in magnesium (**A**), calcium (**B**), sodium (**C**), and potassium (**D**) content of date syrup-fortified laban (DSL), positive control (PC), and negative control (NC) drinks during cold storage at 4 °C for 7 days.

The results of mineral content in laban samples (NC, PC, and DSL) as affected by storage period are shown in Figure 3. Incorporation of date syrup in laban drink significantly improved (*p* < 0.05) the magnesium content, as the amounts of magnesium in DSL samples were almost double that in NC and PC (Figure 3A), indicating the improved nutritional and health quality attributes of laban flavored with date dibs [47]. Magnesium is a highly important mineral in health and nutrition, as it has essential roles in the physiological activity of the heart, brain, and muscles, is a cofactor in various enzymes, and is involved in the synthesis of nucleic acids [48]. In agreement with this finding, previous studies indicated that date dibs increased the magnesium content of fermented dairy products [33,49]. In addition, the increment in magnesium content following the addition of dates was observed in fermented milk beverages [35] and yogurt [50]. The magnesium content of all samples fluctuated during storage, and the highest value was observed in the DSL drink at the end of storage period, which could be due to the liberation of magnesium during the degradation of proteins and phenolic compounds by the action of the fermenting microorganism [51]. Similarly, an increase in magnesium content during storage of bio-yogurt fortified with date syrup was reported [49].

Incorporation of a date syrup and sugar mixture in laban drinks slightly reduced the calcium content of the product (Figure 3B). This is comparable with a previous report that showed a slight effect of dates on the calcium content of some fermented milk products [33,34,49]. Laban drink samples containing date syrup showed low calcium content compared with NC and PC samples. This is likely due to the presence of antinutritional factors such as tannin and phytates in date syrup [31,46] that can form complexes with calcium and thereby reduced calcium extractability and quantification ability [52]. During the storage, the calcium content of all samples fluctuated with a general increase in NC and DSL samples and a general reduction in PC samples; however, it remained within the range of 700 to 900 ppm, comparable with that reported in different types of yogurt [53].

The results also show differences in the sodium content between NC, PC, and DSL drinks (Figure 3C). The addition of date syrup increased (*p* < 0.05) the sodium content in fresh laban drink; however, during storage, except for the fifth day, the sodium content of DSL drink was lower than that in both the NC and PC drinks. The low sodium content in the DSL drink could be due to the presence of chelating polyphenols such tannins and phytates that captured sodium and thereby reduced its extraction and subsequent quantification processes [31,46,52]. Previous reports showed similar findings on the sodium content in fermented and flavored dairy products [54,55].

The addition of date syrup and sugar solution to laban drink affected the potassium content in different ways, and the content of potassium was generally higher in the PC drink than that in DSL and NC drinks (Figure 3D). There were slight changes in potassium content during storage, with a general irregular fluctuation. The findings of this study are in agreement with previous reports indicating that the addition of dates improved the potassium content of fermented dairy products [33,34,38,49]. In addition, the amount of potassium in this study was comparable with amounts (921–1337 mg/kg) reported in yogurt fortified with fruit [54]. The incorporation of date syrup and sugar solution in laban drink did not affect the total mineral content of the product; however, the storage time affected minerals in a different manner (data not shown). It has been noted that total mineral levels were higher in laban drink samples containing dibs or sugar solution than that in the negative control. During storage, the total mineral content of laban drink containing sugars and date syrup was reduced, whereas, that of NC showed a slight increase. The differences in the effect of storage on the total minerals content of laban drink samples could be due to variations in the type and activity of fermenting microbes and the fermentation products [56].

### 3.6. Microbial Load of Date Syrup-Fortified Laban Drink (DSL) during Cold Storage

**Figure 4:**
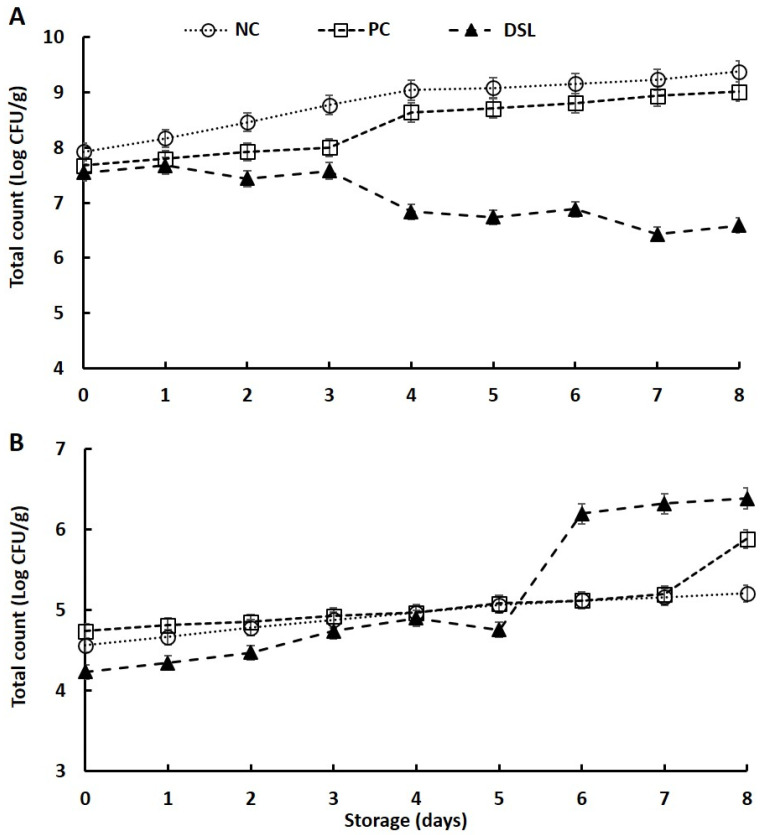
Changes in total bacterial (**A**) and total yeast (**B**) counts of date syrup-fortified laban (DSL), positive control (PC), and negative control (NC) drinks during cold storage at 4 °C for 7 days.

The results on the effect of the addition of date syrup and sugar solution on the microbial load of laban are shown in Figure 4. It is clear that the addition of date syrup and sugar solution significantly reduced (*p* < 0.05) the total microbial counts as compared with that of NC, as the microbial loads of DSL and PC drinks were lower than that of NC at the first day of storage (Figure 4A). This finding is in line with the preservation effects of solution containing high levels of sugars such as date syrup through the reduction in water activity [57]. During the storage, NC and PC drinks showed a significant increase (*p* < 0.05) in the total microbial counts, whereas the DSL drink showed a significant reduction (*p* < 0.05), suggesting that date syrup exhibited antimicrobial effects in laban drink. The reduction in total counts in DSL drink during storage could be attributed to secondary metabolites such as phenolic compounds, glycoproteins, and polypeptides that formed during the degradation of syrup components by fermenting microbes [46,58]. Several reports indicated that date syrup contains appreciable amounts of phenolic compounds, having antimicrobial activity [46,58]. The total microbial counts in this study were comparable with those reported in traditionally produced yogurt [59], fermented milks [60,61], and fermented milk fortified with wheat germ [62]. The counts of yeasts and molds were affected by both the addition of date syrup and by storage conditions. The yeasts and molds counts were low in the DSL drink at the beginning of storage due to the inhibition effect of date syrup, as it has antimicrobial activity [46,58]. During storage, the number of yeasts and molds were significantly increased (*p* < 0.05), with the highest increment being in DSL, followed by NC and then PC (Figure 4B). The increase in yeasts and molds during storage of laban drink was likely due to increased activity of osmophilic yeast and molds that prefer medium with low water activity [57]. The findings on yeasts and molds in this study are also comparable with those reported previously in various fermented dairy products [59,60,61,63,64,65,66]. 

### 3.7. Sensory Evaluation of Date Syrup-Fortified Laban Drink (DSL) during Cold Storage

**Figure 5:**
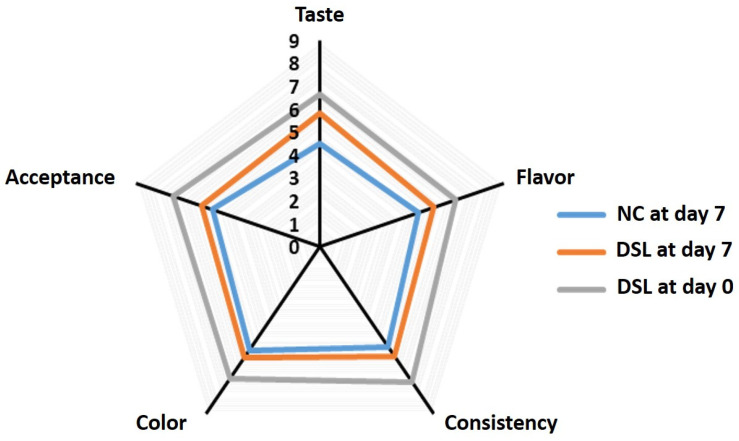
Sensory evaluation of DSL drink at day 7 of storage compared with that of NC laban drink at day 7 of storage and DSL drink at day 0 of storage. NC, negative control; DSL, date syrup-fortified laban.

Sensory evaluation was conducted for DSL drink at day 0 and day 7 of storage in comparison with that negative control (NC) at the seventh day of storage. Fresh DSL drink showed the highest scores on all sensory attributes (Figure 5). At the end of the storage period (7 days), DSL drink exhibited higher scores (*p* < 0.05) on all sensory attributes than the NC drink, indicating that incorporation of date syrup in laban beverage enhanced the sensory quality of the product. In addition, date syrup also preserved (*p* < 0.05) the sensory attributes of laban drink after 7 days of storage. The enhancement of sensory attributes of DSL drink is likely due to the sweet taste and flavor of date syrup, whereas the improvement in the storage stability of the DSL drink could be due to the high sugar content of date syrup, which reduced the water activity of the developed product and thereby eliminated the growth of spoilage microbes. In agreement with these findings, fortification of fermented dairy products with dates significantly improved their sensory attributes compared with non-fortified samples [34,35,49,50,67]. Overall, the findings of this study indicate that fortification of laban with date syrup enhanced the sensory acceptability of this product and hence can promote the large-scale production, trading, and consumption of this dairy beverage.

## 4. Conclusions

Utilization of surplus milk and date syrups produced in Saudi Arabia to produce a natural, sweet, healthy, and high nutritional value dairy drink was the main aim of this study. In this sense, the nutritional, microbial, and organoleptic quality attributes of the developed drink were assessed during cold storage for 1 week. The results reveal that the addition of 12.5% date syrup to fermented milk (laban) improved the nutritional quality, as it increased the levels of ash, protein, total solids, fructose, glucose, and magnesium of the developed drink. Incorporation of date syrup in laban drink also controlled the microbial quality of the product and improved the sensory quality by reducing the acidity and increasing the taste and overall acceptability of the developed product. Moreover, date syrup also acted as preservative in laban drink and maintained the nutritional, microbial, and sensory quality properties of the product during cold storage for 7 days. Overall, this innovative new flavored laban drink can be of high nutritional and health value and could attract consumers, as it did not contain artificial additives, refined sweeteners, and preservatives.

## Figures and Tables

**Table 1:** Physicochemical composition of date dibs and laban (fermented milk).

Parameters	Mean ± SD
Date syrup	
Moisture (g/100 g)	24.89 ± 0.59
Protein (g/100 g)	0.88 ± 0.01
Fat (g/100 g)	0.10 ± 0.00
Fiber (g/100 g)	0.10 ± 0.00
Ash (g/100 g)	1.49 ± 0.02
Total sugars (g/100 g)	53.05 ± 0.95
Glucose (g/100 g)	26.51 ± 0.10
Fructose (g/100 g)	26.54 ± 0.01
Sucrose (g/100 g)	0.10 ± 0.00
Maltose (g/100 g)	0.10 ± 0.01
Calcium (mg/L)	424.00 ± 2.00
Sodium (mg/L)	164.00 ± 1.00
Potassium (mg/L)	0.54 ± 0.01
Magnesium (mg/L)	420.0 ± 1.96
Vitamin A (U/100 g)	1.00 ± 0.02
Vitamin D (U/100 g)	10.00 ± 0.30
Soluble solids (°Bx)	71.11 ± 0.11
pH	5.12 ± 0.13
Water activity	0.71 ± 0.05
Acidity (mg lactic acid/L)	1.00 ± 0.01
Energy (Kcal/100 g)	216.62 ± 2.00
L*	6.89 ± 0.05
a*	2.23 ± 0.11
b*	7.59 ± 0.08
Laban	
Protein (g/100 g)	3.12 ± 0.01
Casein (g/100 g)	2.85 ± 0.01
Fat (g/100 g)	3.20 ± 0.02
Ash (g/100 g)	0.79 ± 0.01
Acidity (% expressed as lactic acid)	0.69 ± 0.00
Total soluble solids (g/100 g)	13.98 ± 0.02
Calcium (mg/L)	726.00 ± 0.75
Magnesium (mg/L)	80.24 ± 0.75
Sodium (mg/L)	305.40 ± 0.75
Potassium (mg/L)	933.00 ± 20.0
pH	4.77 ± 0.02
Bx	7.11 ± 0.01
Moisture (%)	87.12 ± 0.13
Water activity	0.995 ± 0.01
L*	95.82 ± 1.12
a*	−2.45 ± 0.45
b*	10.57 ± 1.11

**Table 2:** Chemical composition of laban drinks during cold storage.

Component	Storage (Day)	NC	PC	DSL
Ash				
	0	0.670 ^(B,e)^ ± 0.005	0.613 ^(C,e)^ ± 0.005	0.741 ^(A,e)^ ± 0.005
	1	0.672 ^(B,e)^ ± 0.006	0.617 ^(C,e)^ ± 0.006	0.743 ^(A,e)^ ± 0.006
	2	0.660 ^(C,b)^ ± 0.005	0.680 ^(B,b)^ ± 0.006	0.740 ^(A,b)^ ± 0.006
	3	0.671 ^(C,b)^ ± 0.001	0.691 ^(B,b)^ ± 0.002	0.751 ^(A,b)^ ± 0.002
	4	0.641 ^(C,d)^ ± 0.001	0.661 ^(B,d)^ ± 0.005	0.721 ^(A,d)^ ± 0.005
	5	0.670 ^(C,bc)^ ± 0.006	0.687 ^(B,bc)^ ± 0.006	0.747 ^(A,bc)^ ± 0.006
	6	0.671 ^(C,bc)^ ± 0.015	0.687 ^(B,bc)^ ± 0.005	0.747 ^(A,bc)^ ± 0.005
	7	0.680 ^(C,a)^ ± 0.011	0.703 ^(B,a)^ ± 0.033	0.760 ^(A,a)^ ± 0.033
Protein				
	0	3.07 ^(B,c)^ ± 0.001	3.05 ^(C,c)^ ± 0.005	3.11 ^(A,c)^ ± 0.005
	1	3.09 ^(B,c)^ ± 0.001	3.07 ^(C,c)^ ± 0.006	3.13 ^(A,c)^ ± 0.002
	2	3.17 ^(B,a)^ ± 0.002	3.15 ^(C,a)^ ± 0.006	3.21 ^(A,a)^ ± 0.006
	3	3.17 ^(B,a)^ ± 0.001	3.15 ^(C,a)^ ± 0.002	3.21 ^(A,a)^ ± 0.002
	4	3.08 ^(B,c)^ ± 0.001	3.06 ^(C,c)^ ± 0.005	3.12 ^(A,c)^ ± 0.005
	5	3.09 ^(B,c)^ ± 0.001	3.07 ^(C,c)^ ± 0.006	3.13 ^(A, c)^ ± 0.003
	6	3.13 ^(B,b)^ ± 0.001	3.11 ^(C,b)^ ± 0.005	3.18 ^(A,b)^ ± 0.005
	7	3.04 ^(B,d)^ ± 0.006	3.02 ^(C,d)^ ± 0.003	3.08 ^(A,d)^ ± 0.004
Total solids				
	0	12.44 ^(C,b)^ ± 0.001	13.41 ^(B,b)^ ± 0.005	19.97 ^(A,b)^ ± 0.005
	1	11.51 ^(C,c)^ ± 0.003	12.49 ^(B,c)^ ± 0.006	19.05 ^(A,c)^ ± 0.002
	2	11.77 ^(C,c)^ ± 0.002	12.74 ^(B,c)^ ± 0.001	19.31 ^(A,c)^ ± 0.001
	3	11.63 ^(C,c)^ ± 0.001	12.60 ^(B,c)^ ± 0.002	19.17 ^(A,c)^ ± 0.002
	4	11.62 ^(C,c)^ ± 0.003	12.59 ^(B,c)^ ± 0.005	19.16 ^(A,c)^ ± 0.001
	5	11.97 ^(C,cb)^ ± 0.001	12.94 ^(B,cb)^ ± 0.006	19.51 ^(A,cb)^ ± 0.003
	6	12.99 ^(C,a)^ ± 0.001	13.96 ^(B,a)^ ± 0.005	20.23 ^(A,a)^ ± 0.005
	7	11.91 ^(C,cb)^ ± 0.006	12.88 ^(B,cb)^ ± 0.003	19.45 ^(A,cb)^ ± 0.004
Fat (%)				
	0	3.13 ^(B,a)^ ± 0.00.	3.10 ^(C,a)^ ± 0.005	2.846 ^(A,a)^ ± 0.005
	1	3.03 ^(B,d)^ ± 0.003	3.00 ^(C,d)^ ± 0.001	2.762 ^(A,d)^ ± 0.002
	2	3.03 ^(B,b)^ ± 0.002	3.00 ^(C,b)^ ± 0.001	2.817 ^(A,b)^ ± 0.001
	3	2.93 ^(B,c)^ ± 0.001	2.90 ^(C,c)^ ± 0.002	2.791 ^(A,c)^ ± 0.002
	4	2.93 ^(B,e)^ ± 0.003	2.90 ^(C,e)^ ± 0.005	2.693 ^(A,e)^ ± 0.001
	5	2.83 ^(B,e)^ ± 0.001	2.80 ^(C,e)^ ± 0.003	2.682 ^(A,e)^ ± 0.003
	6	2.83 ^(B,cd)^ ± 0.001	2.80 ^(C,cd)^ ± 0.002	2.772 ^(A,cd)^ ± 0.005
	7	2.73 ^(B,f)^ ± 0.002	2.70 ^(C,f)^ ± 0.003	2.595 ^(A,f)^ ± 0.004
Acidity				
	0	0.737 ^(C,f)^ ± 0.001	0.753 ^(B,f)^ ± 0.001	0.689 ^(A,f)^ ± 0.005
	1	0.747 ^(C,f)^ ± 0.003	0.767 ^(B,f)^ ± 0.001	0.702 ^(A,f)^ ± 0.002
	2	0.873 ^(C,e)^ ± 0.002	0.890 ^(B,e)^ ± 0.001	0.826 ^(A,e)^ ± 0.001
	3	0.883 ^(C,e)^ ± 0.001	0.900 ^(B,e)^ ± 0.002	0.837 ^(A,e)^ ± 0.002
	4	1.003 ^(C,d)^ ± 0.003	1.021 ^(B,d)^ ± 0.005	0.956 ^(A,d)^ ± 0.001
	5	1.173 ^(C,c)^ ± 0.001	1.193 ^(B,c)^ ± 0.003	1.127 ^(A,c)^ ± 0.003
	6	1.297 ^(C,a)^ ± 0.001	1.311 ^(B,a)^ ± 0.002	1.249 ^(A,a)^ ± 0.005
	7	1.217 ^(C,b)^ ± 0.006	1.233 ^(B,b)^ ± 0.003	1.170 ^(A,b)^ ± 0.004
Casein				
	0	2.787 ^(B,a)^ ± 0.00.	2.700 ^(C,a)^ ± 0.005	2.846 ^(A,a)^ ± 0.005
	1	2.683 ^(B,d)^ ± 0.003	2.617 ^(C,d)^ ± 0.001	2.762 ^(A,d)^ ± 0.002
	2	2.737 ^(B,b)^ ± 0.002	2.670 ^(C,b)^ ± 0.001	2.817 ^(A,b)^ ± 0.001
	3	2.713 ^(B,c)^ ± 0.001	2.643 ^(C,c)^ ± 0.002	2.791 ^(A,c)^ ± 0.002
	4	2.613 ^(B,e)^ ± 0.003	2.547 ^(C,e)^ ± 0.005	2.693 ^(A,e)^ ± 0.001
	5	2.600 ^(B,e)^ ± 0.001	2.533 ^(C,e)^ ± 0.003	2.682 ^(A,e)^ ± 0.003
	6	2.690 ^(B,c,d)^ ± 0.001	2.623 ^(C,cd)^ ± 0.002	2.772 ^(A,cd)^ ± 0.005
	7	2.513 ^(B,f)^ ± 0.002	2.447 ^(C,f)^ ± 0.003	2.595 ^(A,f)^ ± 0.004

Values are mean of triplicate determinations (*n* = 3) ± standard deviation. Means followed by different capital letter within the same row (treatments) or different small letter within the same column (storage time) are significantly different at *p* < 0.05. NC, negative control (non-flavored laban drink); PC, positive control (laban drink flavored with sugar solution of glucose and fructose); DSL, laban drink flavored with 12.5% date syrup.

## Data Availability

The datasets generated for this study are available on request to the corresponding author.
